# The Biosynthesis of Infrared-Emitting Quantum Dots in *Allium Fistulosum*

**DOI:** 10.1038/srep20480

**Published:** 2016-02-09

**Authors:** M. Green, S. J. Haigh, E. A. Lewis, L. Sandiford, M. Burkitt-Gray, R. Fleck, G. Vizcay-Barrena, L. Jensen, H. Mirzai, R. J. Curry, L.-A. Dailey

**Affiliations:** 1 Department of Physics, King’s College London, The Strand, London WC2R 2LS, UK; 2 School of Materials, The University of Manchester, Manchester, M13 9PL, UK; 3 Department of Imaging Chemistry and Biology, Divisions of Imaging Science and Biomedical Engineering, King’s College London, 4^th^ floor, Lambert Wing, St Thomas’ Hospital, London SE1 7EH, UK; 4 The Centre for Ultrastructural Imaging, King’s College London, New Hunt’s House, London SE1 1UL. UK; 5 Advanced Technology Institute, Department of Electronic Engineering, University of Surrey, Guildford, Surrey GU2 7XH, UK; 6 Institute of Pharmaceutical Science,, King’s College London, 5^th^ Floor, Franklin-Wilkins Building, 150 Stamford Street, London SE1 9NH, UK

## Abstract

The development of simple routes to emissive solid-state materials is of paramount interest, and in this report we describe the biosynthesis of infrared emitting quantum dots in a living plant *via* a mutual antagonistic reaction. Exposure of common *Allium fistulosum* to mercury and tellurium salts under ambient conditions resulted in the expulsion of crystalline, non-passivated HgTe quantum dots that exhibited emissive characteristics in the near-infrared spectral region, a wavelength range that is important in telecommunications and solar energy conversion.

The emergence of the high temperature, inert-atmosphere organometallic route to quantum dots in the 1990s presented a landmark method to make most semiconductors on the nanoscale^1^. The optimisation of reaction conditions allowed the growth of highly structured materials with controllable electronic and optical properties using relatively simple synthesis techniques. Simultaneously, aqueous routes towards colloidal semiconductors emerged yielding high quality thiol-capped CdTe quantum dots prepared without the need for a further inorganic shell^2^. Both these routes gave highly luminescent nanomaterials in differing solvent and chemical environments. The need to further simplify these synthetic pathways (which utilise inert atmospheres and some degree of heating) has led to the emergence of biosynthesis, combining aqueous colloidal chemistry and existing natural biological processes to produce nanostructured solid-state materials^3^. We previously reported the synthesis of CdTe quantum dots in the chloragogenous cells of a live worm, exploiting a reaction known as mutual antagonism to produce the nanomaterials as a by-product against exposure to heavy metals^4^. Mutual antagonism has been known to toxicologists since the 1960s^5^, and has been evoked to explain the formation of inorganic granules in a variety of living animals after exposure to mercury and selenium compounds^6^. Although well-known to toxicologists, few materials scientists are aware of the biological process where two precursors react to form a less-toxic species (which coincidentally happens to be a semiconductor), analogous to numerous reactions in materials chemistry. In the case of mercury exposure, a large number of metazoans (especially higher trophic marine animals) respond to such a threat by reacting the toxic metal ion with selenium and selenoprotein P, producing tiemannite (HgSe) inorganic granules, thereby locking-in the toxic elements in a relatively non-toxic form^7^,^8^. Mutual antagonism has also been reproduced in plants by the addition of a chalcogen precursor with the toxic metal cation, mimicking the formation of tiemannite granules in large animals^9^,^10^. This process has the advantage of being easier to manipulate into a routine synthetic procedure for material synthesis. Whilst HgSe is a known narrow band gap semiconductor, the optical properties are inferior to the related HgTe in quantum dot form^11^, notably when capped with a thiol species, in a similar manner to CdTe as mentioned above^2^. In this report, we demonstrate the first direct production of technologically relevant infra-red emitting semiconductor HgTe quantum dots, via mutual antagonism in living plants.

In a typical reaction, *Allium fistulosum* (common spring onion) were obtained from commercial sources and grown for a further month in ambient conditions. To the plant’s growth solution were then added equimolar amounts of Hg(O_2_CCH_3_)_2_ and Na_2_TeO_3_. The plants were then allowed to grow for a further seven days at which point the plants were removed from the growth solution. The roots of the plants which had been exposed to the precursors had a black coating, unlike control samples which showed no evidence of solid-state material growth (Fig. 1A). The black material could then be mechanically removed using a spatula, and dispersed in water with sonication, forming a suspension, or left *in situ* on the plant root.

Electron microscopy of the dispersion showed polydispersed nanoparticles, up to 20 nm in diameter (Fig. 1B). High-resolution transmission electron microscopy (HRTEM) again showed discrete non-spherical, single crystal particles with distinct facets (Fig. 1C) and the Fourier transform confirmed the expected reflections consistent with HgTe oriented along [100] (Fig. 1D). The non-spherical shape of these nanoparticles is in agreement with other reports of Hg chalcogenide quantum dots, where a variety of morphologies is routinely reported by varying the synthesis temperature, which is usually significantly lower than other II–VI semiconductors. This has been attributed to the positive redox potential for Hg^2^, which makes the reduction step of the group II precursor favourable^12^.

Atomic resolution high-angle annular dark field (HAADF) scanning transmission electron microscopy (STEM) of the sample showed non-spherical, single crystal particles with multiple twinning planes, the identity of which again was confirmed by indexing the measured d-spacings to the lattice spacings of mercury telluride (HgTe) (Fig. 2 and supporting information).

In some cases, several crystals approximately 5 nm in diameter appeared to have aggregated, consistent with materials with no surface capping agents (Fig. 3). Energy dispersive X-ray spectroscopy (EDXS) analysis of individual particles showed the presence of mainly Hg and Te in ratios of approximately 1:1, surprisingly with no associated carbon or sulfur (Fig. 3C) which suggested the absence of a surface passivating ligand, unlike CdTe dots prepared previously by biosynthesis which showed evidence of a small-molecule thiol-based capping agent^4^. The presence of a thiol capping agent is beneficial in some nanoscale semiconductors, notably CdTe and HgTe, where the coordinating functional group is the basis for the observed enhanced emission due to the alignment of energy levels of both the surface ligand and semiconductor leading to blocked hole trapping states^13^,^14^. Whilst a surface capping layer is essential for specific applications such as biological imaging, it does, however, present a potential barrier for charge carriers. Other applications, such as solar energy conversion, would benefit from the absence of a passivating layer to remove any obstacle to charge carrier separation, or to provide a clean surface to attach a specifically engineered ligand^15^. An electron microscope investigation into the biological processes in the plant with regards to where the reactions occurred was also conducted (Fig. 4). The *Allium* root was examined after 7 days exposure to the precursors, specifically the sections localised through the apical meristem, the elongation zone and the basal zone just beneath the bulb crown where the roots looked particularly blackened. Inclusions were primarily observed in the cortex at the division zones near the meristem. In the sections within the elongation zone, the inclusions appeared to be mainly inside the large central vacuole of the cortical cells. The presence of inorganic inclusions was also observed on the cell wall of the roots in sections near the crown. EDXS analysis of the inclusions identified Hg although Te was only marginally detected in contrast to the analysis of the particles dispersed in solution described above. This was explained by the presence of uranium peaks, associated with the uranyl acetate stain introduced during processing to enhance TEM image contrast, which overlapped with the tellurium signal.

Whilst the reaction is based upon the aqueous route with regards to choice of target material and precursors, this synthesis is notably different and can be considered a ‘green’ alternative for a number of reasons; the actual mechanism suggested here (mutual antagonism) is a natural process that has evolved in plants and animals to specifically reduce the impact of heavy metals. No organic solvents, capping agents or extraneous precursors were utilised unlike bench-based organometallic routes, nor was heating required or the presence of inert gases, all of which require processing and the expenditure of resources. The mechanisms involved in particle formation were clearly driven by the inherent biological processes in the plant, such as the reduction of the Te^IV^ species to Te^II^, which is essential to form the materials reported. Similar processes have been highlighted in the plant-based reduction of arsenate species to arsenite by enzymes^16^. The exact mechanism behind the formation of HgTe quantum dots is unclear; whilst HgSe granules have been noted in the literature, their formation in marine animals have in most cases been linked to selenoprotein P, which is not present in the plant used in this study. Likewise, the mechanism we suggested in our previous work on CdTe synthesis in earthworms is unlikely to be applicable. Studies by Caruso^9^ on the formation of tiemannite in *Allium* highlighted numerous possible mechanisms due to the observation of Hg-Se, Hg-only and Se-only regions in the plant, with the conversion of Na_2_SeO_3_ to methylselenocysteine being a predominant reaction in the reduction of selenium toxicity. Whilst there are few reports on tellurium metabolites in plants, *Te*-methyltellurocysteine oxide has been observed as the major metabolic species from the exposure of *Allium sativum* to Na_2_TeO_4_. *Te*-methyltellurocysteine oxide was further reported to degrade to either methyltellurol or methyltellurous acid, which was then sequestered by cysteine giving *S*-methyltellurylsulfide^17^. Assuming a similar reaction in *Allium fistulosum*, which of these metabolites is the actual tellurium precursor is at present unknown although methyltellurol has previously been reported as a precursor in the preparation of CdTe and HgCdTe thin films by chemical vapour deposition^18^. It is also worthy to note that alkyltellurols (RTeH; R = alkyl) have been shown to react with group II metal species to give the family of compound M(TeR)_2_ (M = Cd, Hg, Zn; R = alkyl group), of which Hg(TeBu)_2_ has been shown to be an effective room-temperature, photolytic precursor to HgTe nanoparticles^19^.

The optical properties of the particles were also examined. Previously, thiol-capped HgTe prepared by aqueous routes under an inert atmosphere have displayed broad, strong emission in the near-infrared region, between 800–1400 nm^14^, with the onset of absorption appearing slightly blue shifted to the emission maxima, usually without a well-defined excitonic peak. In the work reported here, we examined both the solution of dispersed particles and the root (which included the biosynthesised particles themselves). The absorption of the HgTe solution reported here showed a broad near-infrared band edge containing a feature consistent with an excitonic peak at *ca.* 1140 nm (Fig. 5a) that was clearly resolved in the derivative spectra. When the solution was excited (λ_exc._ = 812 nm), broad near-IR emission was observed (Fig. 5b) extending from *ca.* 1000–1375 nm. The reduction in emission intensity at *ca.* 1175 nm and lack of any measurable emission beyond ~1375 nm is a result of the strong absorption of the emission by the aqueous solution. Similar multi-featured emission spectra have been observed previously and are common for HgTe prepared in aqueous solution^20^. The emission reported here was substantially weaker than the thiol-capped HgTe nanoparticles prepared in solution (quantum yield below 1%), however this is unsurprising due to the absence of a thiolated capping agent, whilst the observation of any emission from an uncapped HgTe quantum dot prepared in air is in itself remarkable. Photoluminescence from the solid nanoparticles still attached to the root was also observed after direct excitation at 812 nm, thus providing the emission of the HgTe quantum dots in the absence of water absorption. This reveals the full spectral range of the emission obtained from the HgTe quantum dots (~900 nm to 1675 nm), which significantly included the wavelengths of technological importance to telecommunications (Fig. 5b). The higher energy emission (below 1000 nm) in these spectra originated from the laser diode excitation source as did the narrow peak at 1624 nm (second order grating diffraction of the source). Excitation of either the solution or plant root using 458 nm resulted in bright green emission attributable to chlorophyll, peaking at *ca*. 520 nm, which was significantly narrowed in the solution (Fig. 5c). No emission was observed from the HgTe quantum dots under this excitation wavelength indicating separation of the emitting chromophore from the HgTe and thus preventing any energy transfer processes from taking place (e.g. Förster energy transfer). Though the HgTe quantum dots were able to absorb the chromophore emission conventionally, their concentration was too weak to observe any re-emission in the near-infrared.

In conclusion, we have prepared near-infrared emitting (900 nm–1675 nm) HgTe quantum dots via a mutual antagonistic reaction in living *Allium fistulosum*. The particles, between 5 and 20 nm in diameter were single crystallites with no native organic capping agent. The report may open up other simple, effective pathways to emissive nanomaterials with interesting optical and electronic properties.

## Experimental

### Plant material and co-cultivation with QD precursors

*Allium fistulosum* were sourced from a local supermarket, and soaked in an aqueous solution of 0.0894% w/w 1% naphthylaceitic acid, sodium salt (Baby Bio roota) for one hour. The plants were then bedded in perlite soil and immersed in deionised water in ambient conditions for 4 weeks (2 plants in 500 ml deionised water (18.2 MΩ), with approximately 2 inches of bulb covered by water). After 4 weeks, the plants exhibited clear growth of leaves and roots. Equimolar amounts of precursors (6.3 × 10^−5^ moles each of Hg(O_2_CCH_3_)_2_ and Na_2_TeO_3_) were the added to the water. Following precursors supplementation, the roots were harvested after a further 7 day growth. The resulting inorganic materials could be removed mechanically from the bulbs or were left in place for further investigation.

### Preparation of Allium roots for Electron Microscopy Investigation

For transmission electron microscopy (TEM) analysis, roots were fixed overnight with 4% (w/v) paraformaldehyde, 2% (v/v) glutaraldehyde in 0.05 M phosphate buffer (pH 7.2). After fixation root samples were washed with buffer, incubated in 1% tannic acid for 30 min and post-fixed in 1% (w/v) osmium tetroxide in 0.05 M phosphate buffer (pH 7.2) for 1 hour. Samples were then *en bloc* stained with 1% aqueous uranyl acetate overnight at 4 ^o^C followed by thorough washing before being dehydrated through a graded ethanol series. Root samples were equilibrated with propylene oxide before infiltration with SPURR resin (TAAB) and polymerised at 70 °C for 24 hours. Semithin sections (0.5 μm) were cut using a Reichert-Jung Ultracut E ultramicrotome and stained with toluidine blue to assess general features. Ultrathin sections (50–70 nm) were also prepared, mounted on 150 mesh copper grids and contrasted using uranyl acetate and lead citrate. Samples were examined on a FEI Tecnai 12 transmission microscope operated at 120 kV. Images were acquired with an AMT 16000 M camera. EDS was used to confirm elemental composition of NP.

### Serial Block face imaging

Root samples were fixed, washed and incubated in tannic acid as above. The rest of the protocol was modified to ensure the samples were heavily stained with heavy metals to guaranty the high contrasts and electron conductivity required for serial block face imaging in the scanning electron microscope. Therefore, root pieces were further fixed in 1.5% potassium ferrocyanide: 2% osmium tetroxide in 0.1 M phosphate buffer for 1 h at 4 ^o^C. Tissue was then thoroughly rinsed in distilled water and incubated in 1% aqueous thiocarbohydrazide for 4 min. After further rinsing, the samples were treated with 2% aqueous osmium tetroxide for 30 min, rinsed and en-bloc stained in 1% uranyl actetate overnight at 4 ^o^C. To further enhance contrasts in the samples, one last treatment with Walton’s Lead solution was carried out for 30 min at 60 ^o^C, before proceeding to dehydration in an ethanol series and infiltration with Durcupan ACM resin (Sigma). After embedding and curing, tissue blocks were mounted on Gatan 3View aluminum pins using conductive glue (CircuitWorks Conductive Epoxy) and trimmed accordingly. Before sectioning/imaging, samples were gold coated to increase electron conductivity. The specimens were then placed inside a Jeol field emission scanning electron microscope (JSM-7100F) equipped with a 3View 2XP system (Gatan). For this particular experiment, sections thickness was set at 40 nm (Z resolution). Samples were imaged at 1 kV under high vacuum using a 4096 × 4096 scan rate, which gave a final pixel size of 45 nm.

### Optical Spectroscopy

Absorption spectra of HgTe suspended in water were obtained using a Varian Cary 3000 spectrophotometer. Photoluminescence spectra were obtained using 812 nm laser diode (Thorlabs) or 457 nm Ar-ion (Coherent) sources. The emission was collected and dispersed in a Bentham TMc300 monochromator (600 g/mm or 1200 g/mm grating) and detected using lock-in amplification (Signal Recovery SR7265) and a InGaAs detector (Newport 818-IG) or Si detector (Newport 818-SL). Emission spectra have been corrected for the system response.

### Scanning transmission electron microscopy

STEM imaging was performed using a probe corrected Titan G2 80–200 (S)TEM microscope operated at 200 kV with a beam current of 200 pA and a convergence semi-angle of 18.5 mrad. HAADF imaging was performed with an inner collection semi-angle of 54 mrad. EDXS spectrum images of size 512 × 512 pixels were acquired using the Titan’s Super-X four silicon drift detector system with a 30 us dwell time and a total acquisition time of ~5 minutes. EDXS data was acquired and processed using Bruker Esprit software with quantification performed using the Cliff-Lorimer approach without absorption correction. STEM images were acquired using FEI TIA software and processed using the Image-J software.

## Additional Information

**How to cite this article**: Green, M. *et al.* The Biosynthesis of Infrared-Emitting Quantum Dots in *Allium Fistulosum*. *Sci. Rep.* **6**, 20480; doi: 10.1038/srep20480 (2016).

## Supplementary Material

Supplementary Information

## Figures and Tables

**Figure 1:**
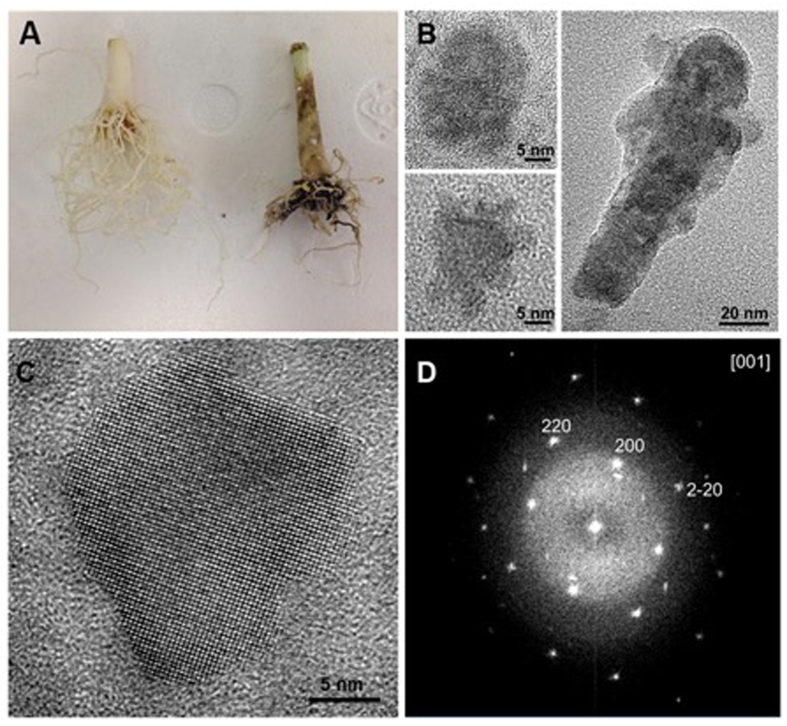
(**A**) Photo of *Allium Fistulosum* roots; left hand side – control with no exposure to precursors, right hand side after 7 days exposure to precursors; (**B**) A selection of transmission electron microscope images of HgTe particles removed from plant root, (**C**) High resolution transmission electron microscope image of a HgTe nanocrystal, (**D**) Fourier transform of the image in (**C**) showing reflections consistent with HgTe oriented along the [001] zone axis.

**Figure 2:**
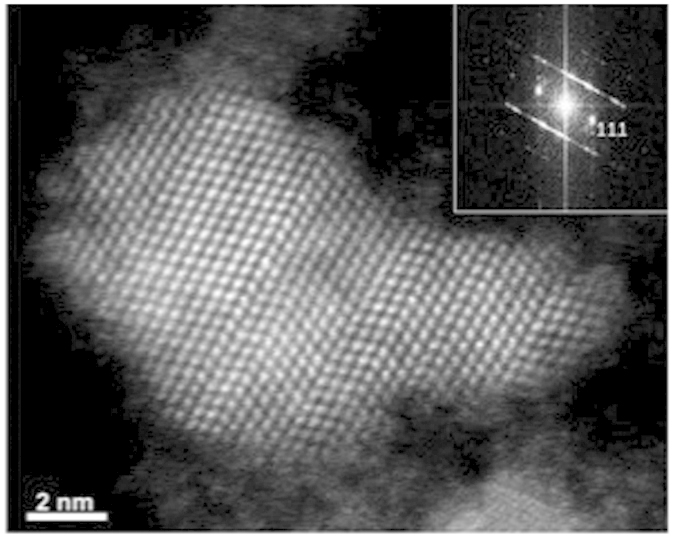
Atomic resolution high angle annular dark field (HAADF) scanning transmission electron microscope (STEM) image of a single highly twinned HgTe quantum dot. Inset; Fourier transforms of the images clearly showing {111} planes of HgTe. An EDX spectrum image of this particle can be found in the supporting information.

**Figure 3:**
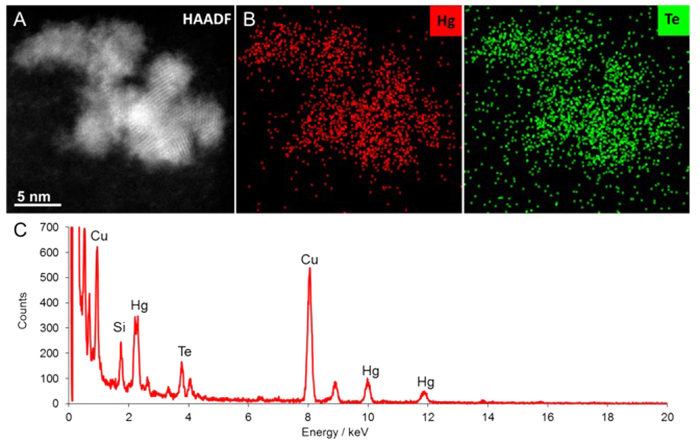
(**A**) High angle annular dark field scanning transmission electron microscope images of HgTe quantum dots. (**B**) Energy dispersive X-ray (EDX) spectrum image of area in (**A**) showing colocalisation of Hg and Te. (**C**) EDX spectra extracted from the full spectrum image in (**B**), EDX quantification reveals a close to stoichiometric composition for HgTe (48 at. % Hg:52 at. % Te).

**Figure 4:**
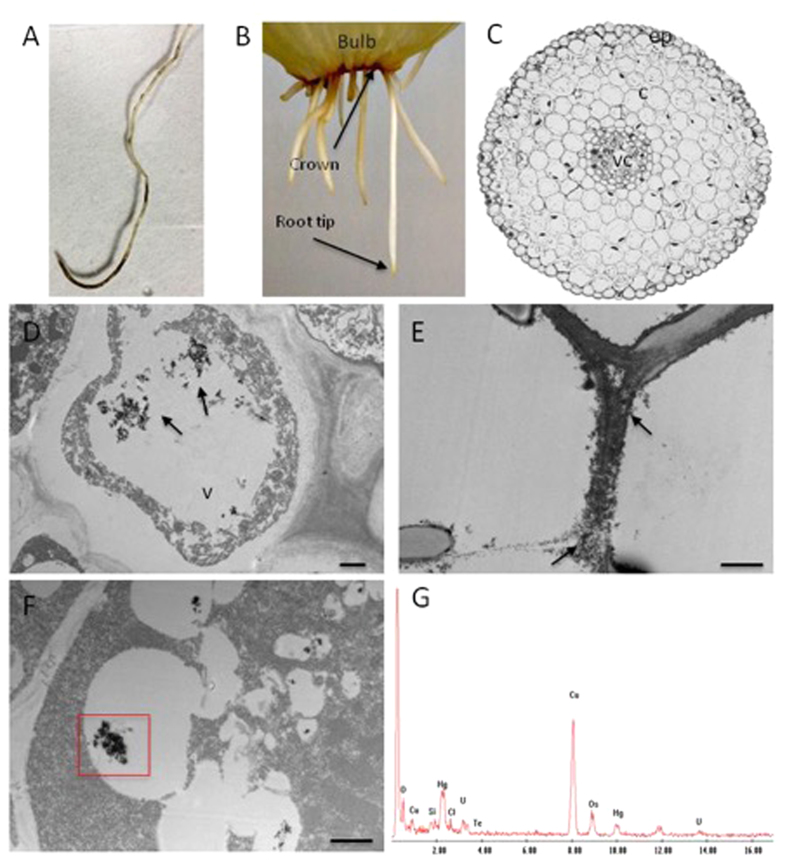
Hg/Te inclusion localization within the root tissues. (**A**) Representative image of a root exposed to Hg/Te salts. Blackened areas are observed all along the root, being more pronounced near the root tip and at the basal zone of the root; (**B**) Samples were collected near the root tip and just below the bulb crown; (**C**). Cross section of main root of *Allium Fistulosum*; epidermis (ep), cortex (c) and vascular cylinder (vc); (**D,E**) Electron micrograph showing inclusions (arrows) in the central vacuole (v) of a cortical cell in D and within the cell wall in (**E–G**) Aggregates accumulate in the vacuole (red box in (**F**) were analysed by EDXS to confirm the presence of Hg and Te as shown in the spectrum (**G**). All scale bars:1 μm.

**Figure 5:**
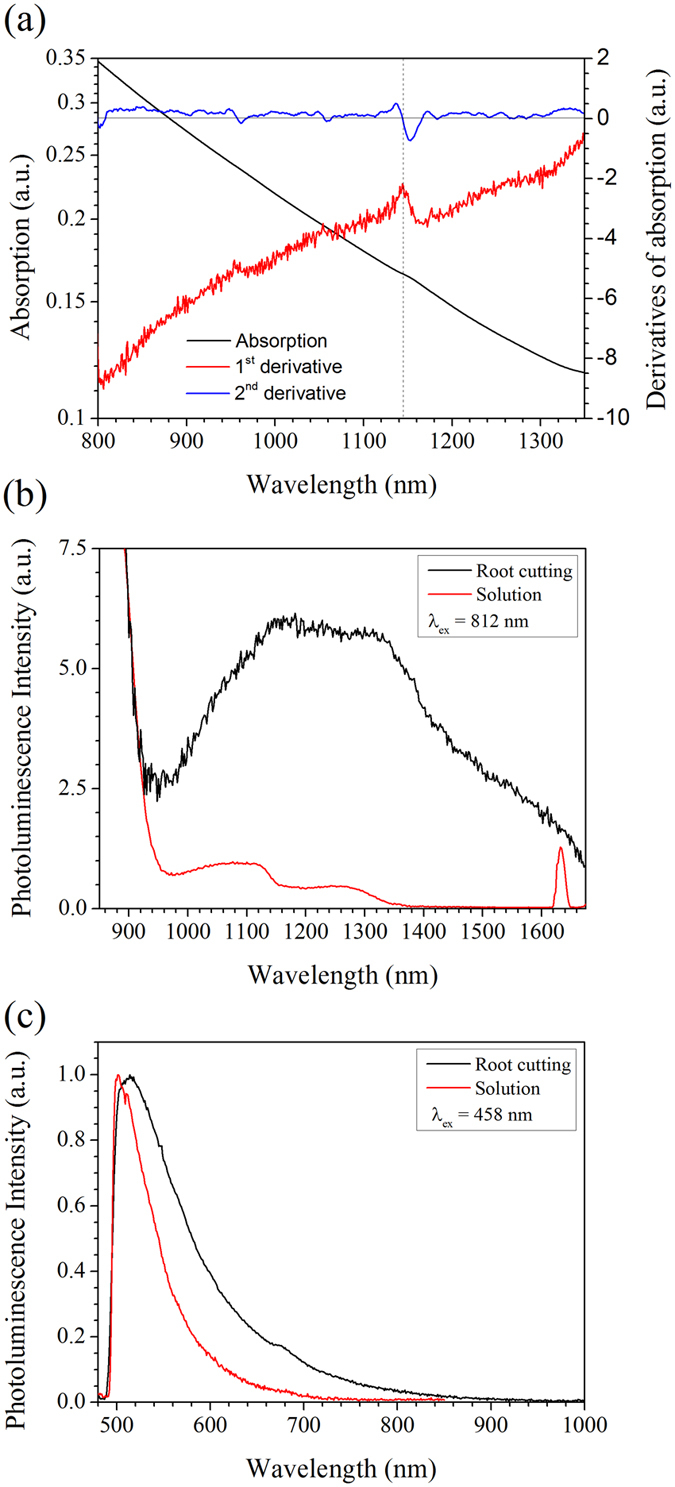
Optical properties of biosynthesised particles. (**a**) Absorption spectrum of an aqueous suspension of HgTe and derivative spectra resolving an excitonic absorption peak at 1140 nm. (**b**) Photoluminescence spectra of an aqueous suspension of HgTe and a root cutting containing solid HgTe product excited at 812 nm revealing broad near-infrared emission. (**c**) Photoluminescence spectra of an aqueous suspension of HgTe and a rout cutting containing solid HgTe product excited at 458 nm displaying strong green emission attributed to chlorophyll.
