# Erratum: The Biosynthesis of Infrared-Emitting Quantum Dots in *Allium Fistulosum*

**DOI:** 10.1038/srep22497

**Published:** 2016-03-04

**Authors:** M. Green, S. J. Haigh, E. A. Lewis, L. Sandiford, M. Burkitt-Gray, R. Fleck, G. Vizcay-Barrena, L. Jensen, H. Mirzai, R. J. Curry, L.-A. Dailey

Scientific Reports 6: Article number: 20480; 10.1038/srep20480 published online: 02092016; updated: 03042016.

The Acknowledgements section in this Article is incomplete.

“S.J.H. and E.A.L. acknowledge funding from the Engineering and Physical Sciences Research Council (EPSRC) UK Grants EP/G035954/1 and EP/J021172/1 and from the Defence Threat Reduction Agency Grant HDTRA1-12-1-0013. E.A.L. would like to thank the North West Nanoscience Doctoral Training Centre (NOWNano DTC) for supporting his studentship. The authors wish to acknowledge the support from H.M. Government (UK) for the provision of the funds for the FEI Titan G2 80-200 S/TEM associated with research capability of the Nuclear Advanced Manufacturing Research Centre.”

should read:

“S.J.H. and E.A.L. acknowledge funding from the Engineering and Physical Sciences Research Council (EPSRC) UK Grants EP/G035954/1 and EP/J021172/1 and from the Defence Threat Reduction Agency Grant HDTRA1-12-1-0013. E.A.L. would like to thank the North West Nanoscience Doctoral Training Centre (NOWNano DTC) for supporting his studentship. The authors wish to acknowledge the support from H.M. Government (UK) for the provision of the funds for the FEI Titan G2 80-200 S/TEM associated with research capability of the Nuclear Advanced Manufacturing Research Centre. Open access for this article was funded by King’s College London.”

