# Supplementary material for: The Biosynthesis of Infrared-Emitting Quantum Dots in Allium Fistulosum
Source: Sci Rep. 2016 Feb 9;6:20480. doi: 10.1038/srep20480 (PMC4746658; doi:10.1038/srep20480)
Supplement: Supplementary Information [file srep20480-s1.pdf]

The Biosynthesis of Infrared-Emitting Quantum Dots in *Allium Fistulosum*.

M. Green,[a]* S.J. Haigh,[b] E.A. Lewis,[b] L. Sandiford,[a,c] M. Burkitt-Gray,[a,d] R. Fleck,[d] G. Vizcay-Barrena,[d] L. Jensen,[d] H. Mirzai,[a] R. J. Curry,[e] L.-A. Dailey.[f]

Supporting information.

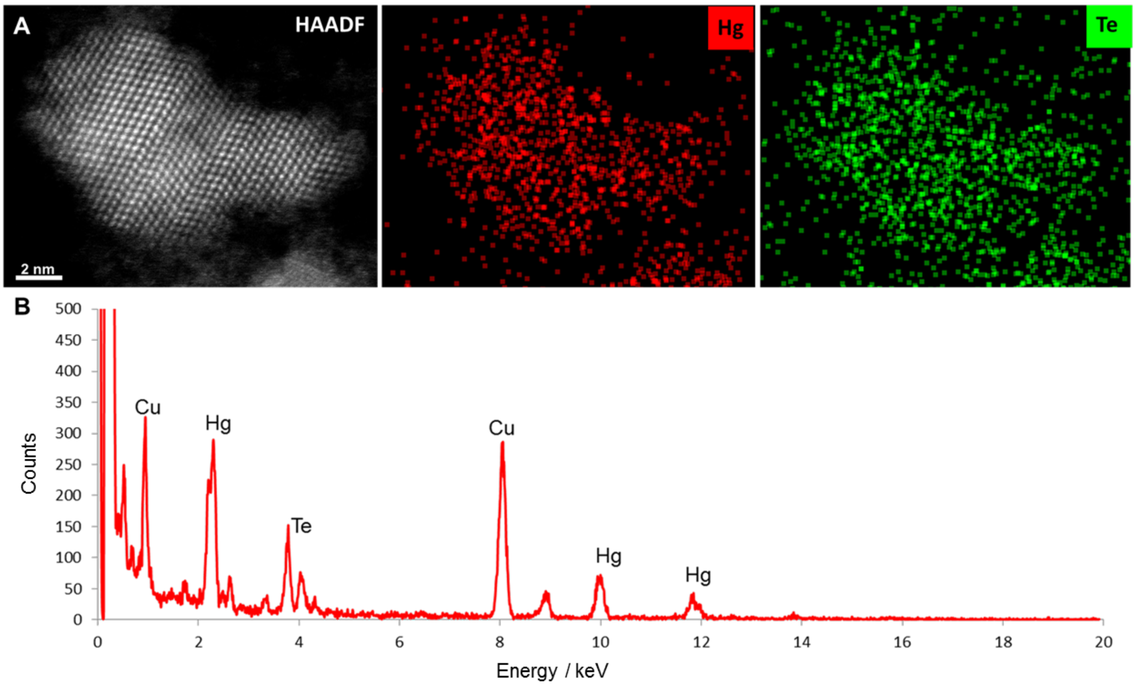

EDX spectrum images and extracted summed spectra for the nanoparticle shown in figure 2.
